# A Systematic Review on Plant-Derived Extracellular Vesicles as Drug Delivery Systems

**DOI:** 10.3390/ijms25147559

**Published:** 2024-07-10

**Authors:** Balázs Kürtösi, Adrienn Kazsoki, Romána Zelkó

**Affiliations:** University Pharmacy Department of Pharmacy Administration, Semmelweis University, Hőgyes Endre Street 7–9, 1092 Budapest, Hungary; kurtosi.balazs@phd.semmelweis.hu (B.K.); kazsoki.adrienn@semmelweis.hu (A.K.)

**Keywords:** plant-derived extracellular vesicles, drug delivery systems, isolation, drug loading

## Abstract

This systematic review offers a comprehensive analysis of plant-derived extracellular vesicles (PDEVs) as emerging drug delivery systems, focusing on original research articles published between 2016 and 2024 that exclusively examine the use of PDEVs for drug delivery. After a rigorous search across multiple databases, 20 relevant studies out of 805 initial results were selected for analysis. This review systematically summarizes the critical data on PDEV components, isolation methods, and drug-loading techniques. It highlights the potential of PDEVs to significantly enhance drug safety and efficacy, reduce dosage and toxicity, and align drug development with sustainable and environmentally friendly biotechnological processes. This review also emphasizes the advantages of PDEVs over mammalian-derived vesicles, such as cost-effectiveness, higher yield, and reduced immunogenicity. Additionally, it explores the synergistic potential between encapsulated drugs and bioactive compounds naturally present in PDEVs. This study acknowledges the challenges in standardizing isolation and formulation methods for clinical use. Overall, this review provides valuable insights into the current state and future directions of PDEV-based drug delivery systems, highlighting their promising role in advancing pharmaceutical research and development.

## 1. Introduction

Some notable challenges mark the current state of pharmaceutical research. While researchers have been involved in developing new small-molecule drugs, these molecules often exhibit a wide range of physicochemical properties. Certain drugs may have poor bioavailability and solubility, which can limit their effectiveness in the human body. Additionally, other drugs may be sensitive to environmental factors such as light, temperature, or the degradative effects of enzymes, which can further limit their utility. 

Nanotechnology provides new exciting research opportunities in many fields. It could be used in IT, the textile industry, the food industry, or medical and pharmacology development. These novel nanosystems have some notable advantages compared to conventional pharmaceuticals [1]. The term nanotechnology originated from the word “nano”, which means “dwarf” in Greek [2]. The U.S. Food and Drug Administration (FDA) classifies particles within the 1–1000 nm size range as nanomaterials if their advantageous physical, chemical, and biological properties are intrinsically linked to their nanoscale dimensions [3]. Synthetic drug delivery systems have been used in recent years to improve drug efficacy and therapeutic effects [4]. The best known of these nano-carrier systems are liposomes, polymer micelles, dendrimers, nanoparticles, nanocrystals, nanosponges, and nanofibers [4,5,6,7]. Nanoscale drug delivery systems have emerged as a promising approach to overcome the limitations of conventional drug formulations. These nanosystems offer several advantages including the following:

Improved drug solubility and bioavailability;

Enhanced cellular uptake and tissue penetration;

Targeted delivery to specific sites of action;

Controlled and sustained drug release;

Protection of sensitive therapeutic agents.

Each has unique characteristics in terms of composition, drug loading capacity, and in vivo behavior. However, challenges remain with some synthetic nanocarriers, such as potential toxicity, immunogenicity, and scalability of production [8].

In recent years, naturally derived nanocarriers have gained interest as potentially safer and more biocompatible alternatives. Among these, plant-derived extracellular vesicles have emerged as a particularly promising platform. PDEVs offer several potential advantages over synthetic nanocarriers. Many drug molecules face limitations that make them good candidates for encapsulation within plant-derived extracellular vesicles. These limitations include poor aqueous solubility, low bioavailability, rapid metabolism and elimination, and an inability to cross biological barriers [2,3]. Hydrophobic drugs with low water solubility can benefit from the lipid bilayer of PDEVs, which can improve their solubility and stability in aqueous environments. Drugs with low bioavailability due to degradation in the gastrointestinal tract or extensive first-pass metabolism can be protected within PDEVs, potentially increasing their absorption and systemic exposure. Additionally, the nanoscale size of PDEVs allows them to penetrate biological barriers more effectively than free drug molecules, potentially improving delivery to target tissues. Encapsulation in PDEVs can also modify the pharmacokinetic profile of drugs, potentially allowing for sustained release and reduced dosing frequency. These properties make PDEVs promising carriers for improving the delivery and efficacy of drugs with inherent physicochemical and pharmacokinetic limitations [9].

**Figure 1:**
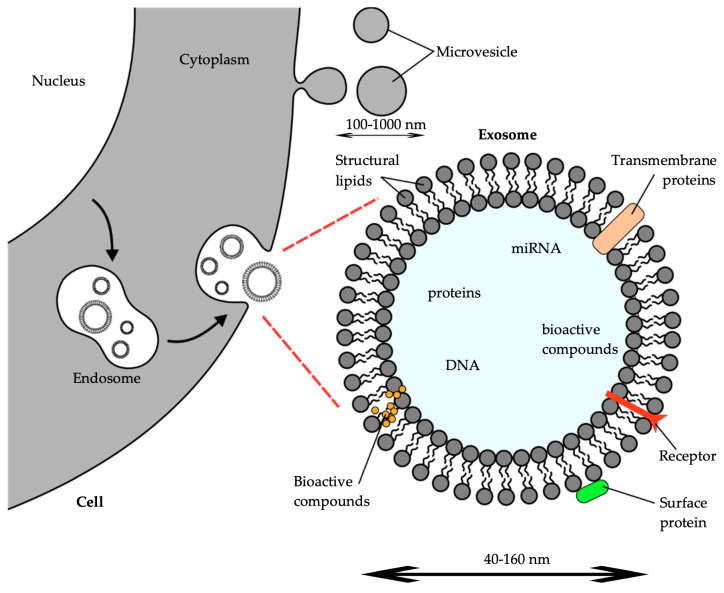
Structure of an exosome released during exocytosis and used as a drug delivery system for external bioactive compounds [12].

The added value of these extracellular vesicles over liposomes are the biocompatibility, the cost-effectiveness, the enhanced drug delivery, and the intrinsic therapeutic properties. The exploration of the biological functions of extracellular vesicles commenced around 1980 [10]. Extracellular vesicles (EVs) are released by all cells, from prokaryotes to eukaryotes. EVs represent a lipid bilayer structure that is crucial in cell-to-cell communication. These EVs can be separated into two groups: ectosomes and exosomes. Ectosomes have arisen from the plasma membrane via outward budding. This group contains macrovesicles, microparticles, and large vesicles of 50 nm to 1 μm in diameter. On the other hand, exosomes are EVs with a range of diameters from 40 to 160 nm. Figure 1 shows an exosome being released from the cell. Depending on the origin of the cell, EVs can contain and deliver a wide range of bioactive compounds, including lipids, proteins, DNA, and RNA [11]. 

PDEVs have more accessible structures and functionality than mammalian extracellular vesicles, presenting a unique advantage. They are significantly more cost-effective and scalable in production, setting them apart from their mammalian counterparts. This distinct characteristic makes plant-derived extracellular vesicles a compelling and practical option for various research and application purposes [13]. The four primary isolation methods for plant-derived vesicles are differential centrifugation [14], sucrose density gradient centrifugation [15], PEG precipitation [16], and enzyme degradation [17]. While differential ultracentrifugation is the most widely used method, all these techniques have pros and cons. They can be combined for better EV outcomes, such as differential ultracentrifugation combined with size exclusion chromatography, which yields better EV purity than the differential ultracentrifugation method [18].

PDEVs naturally contain many bioactive compounds that play an essential role in cell-to-cell communication. This inherent property of PDEVs makes them a potential tool for treating pathological cells or tissues without needing external intervention [19]. It is important to note that while edible fruits typically contain well-known phytochemicals, the composition of these compounds in fruit juice and isolated EVs may differ. Molecules present in fruit or vegetable juice may not be represented in the same composition in EVs [20]. This distinction underscores the unique nature of PDEVs and their potential for use in drug delivery.

Like mammalian EVs, these vesicles have their impact and purpose in intercellular communication, but their cost-effective yields are more efficient. Furthermore, edible plants can also be used as a source of PDEVs. Because we have been consuming these plants since immemorial times, our immune system has developed a natural compatibility with them. The isolation of EVs from plants was successful in many cases, including ginger [21], grape [22], tomato [23], broccoli [24], etc. Recent studies have shown the opportunity to carry different active pharmaceutical ingredients (APIs) in PDEVs as a drug carrier system. PDEVs, as a drug carrier system, are receiving more attention, and for instance, small molecules and external miRNAs [24] and other proteins like HSP70 [25] can be loaded into these vesicles.

The application of EVs as drug delivery vehicles offers multiple benefits. Many studies aim to improve the bioavailability and permeability of different types of APIs through new technological advancements. EVs loaded with drugs can effectively enhance their bioavailability, even allowing them to cross epithelial barriers. This makes EVs particularly valuable for delivering hydrophilic and hydrophobic drugs, thereby broadening therapeutic options for various diseases [14,26].

Encapsulating drug molecules within EVs can also improve their stability. The vesicles’ phospholipid membrane provides a protective effect, shielding sensitive molecules from adverse external conditions, and can lower the drug toxicity [26,27]

Moreover, EVs naturally contain bioactive compounds. However, the specific compounds responsible for their biological effects still require further investigation. Nevertheless, loading an external active substance into an EV can potentially synergize with the vesicle’s inherent active components, leading to enhanced therapeutic outcomes [28]. This interplay between the exogenously added substance and the vesicle’s native constituents may amplify the effectiveness of treatments and offer a promising new opportunity for future medical applications.

These findings suggest that PDEVs offer numerous unexploited possibilities as a drug carrier system, especially for highly potent substances with cytostatic effects, such as Methotrexate, Doxorubicin, Tamoxifen, [18,28,29], etc. The method of loading vesicles largely depends on the active substance and available resources; both active and passive techniques are available. The passive method is driven by diffusion through the vesicle membrane layer into the core. Usually, a few hours of incubation and stirring at room temperature [29] or a higher temperature of 37 °C is enough for encapsulation [30]. This method is simple, and cost- and time-effective, and it does not require special equipment.

Another option is the active method, which uses external energy. EVs were successfully isolated from broccoli and loaded with Astaxanthin using the ultrasound capsulation method [14]. 

Based on the above, it can be stated that the isolation, study, and application of PDEVs as drug delivery systems represent an intriguing journey within pharmaceutical research.

## 2. Results

### 2.1. Database Search and Included Studies 

**Figure 2:**
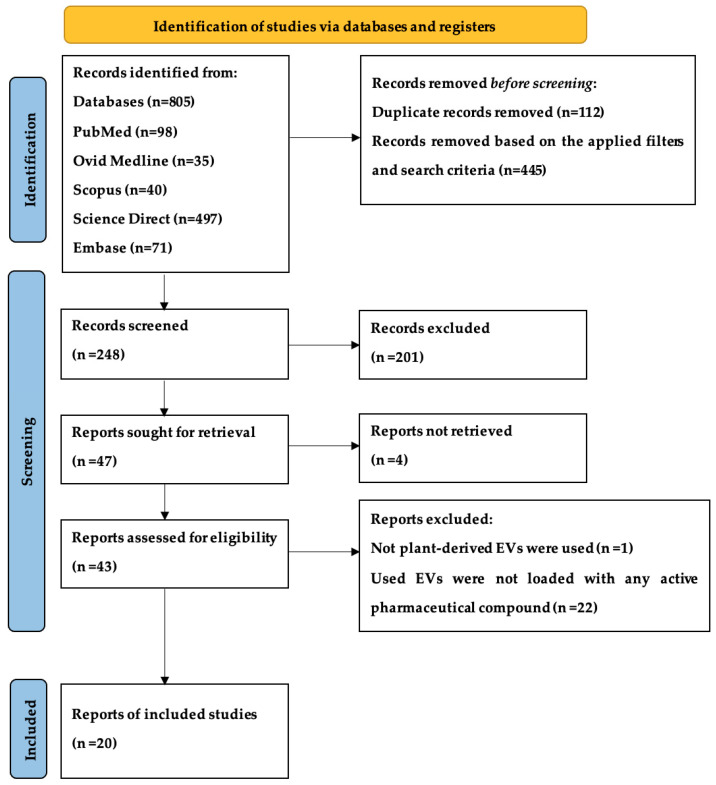
This PRISMA-2020 flow diagram shows the relevant articles included in this study.

A total of 805 articles were retrieved through database searches. Among them, 98 were sourced from PubMed, 35 from Ovid Medline, 40 from Scopus, 497 from Science Direct, 71 from Embase, and 64 from Web of Science. The identification and screening process is outlined in Figure 2.

### 2.2. Results of the Studies 

Based on our filters, all relevant articles containing successfully isolated EVs from certain plants are summarized in Table 1. According to the extracted relevant information, it can be said that PDEVs possess optimal physicochemical properties and an affinity for serving as a drug carrier system. The vesicle sizes ranged from 65 nm to 458 nm, which are optimal for drug loading. The number of particles in the samples ranged from around 10^8^ to 10^12^, which can serve as a guideline for experimental designs.

**Figure 3:**
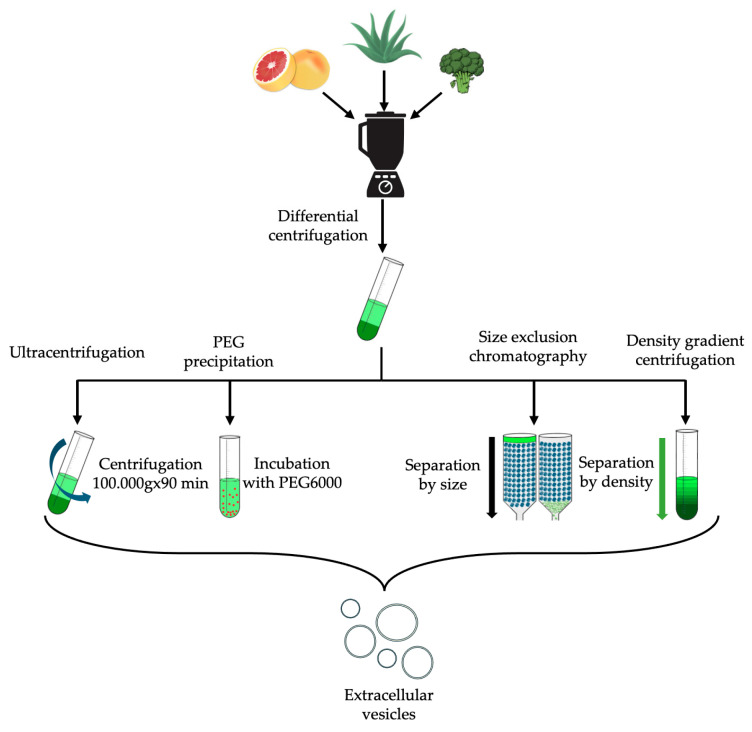
Schematic diagram of isolation methods.

The isolation of EVs from plants faces some challenges. Different types of techniques have been used, but these techniques have limitations. The biggest problem is that none of the methods used are standardized. Therefore, isolated EVs have a wide range in size, purity, and particle number. The isolation methods used to separate plant and mammalian EVs differ slightly. Differential ultracentrifugation (DUC), density gradient centrifugation (DGC), size exclusion chromatography (SEC), PEG precipitation techniques, or a combination were the most employed techniques in the analyzed articles. Therefore, a brief description of these four techniques is provided. A schematic diagram of the isolation methods is shown in Figure 3.

A widely used method is the *ultracentrifugation technique*, but there are no precise specifications as to which speed combination is the most ideal. However, the speed of rotation, the duration of centrifugation, and the type of rotor have a major influence on the quality of the sample. A standard procedure is as follows: firstly, a low speed is applied for 10 to 90 min to remove large EVs and cell debris, followed by maximum speed for 45 to 150 min to recover small EVs [56]. Nevertheless, ultracentrifugation is considered the primary isolation technique when the goal is to produce plant-derived extracellular vesicles [20]. According to studies gathered in 2016, researchers use differential ultracentrifugation in 81% of cases and a combination of techniques in 59% [57].

For example, if researchers want to increase purity, low-speed centrifugation can exclude large particles and cell debris from the sample. Additionally, an SEC step can be added to the procedure for gentle cleaning. Density gradient cleaning is also widely combined with DUC. For this, a different density medium should be prepared, and each density fraction can be separated after centrifugation. Commonly used media for EV isolations are sucrose and iodixanol [58].

The *density gradient technique* relies on the densities of different types of EVs. Gradients are made from various dense mediums containing sucrose, iodixanol, and an appropriate buffer. The density decreases from the bottom to the top. Two methods can be used for EV separation: “bottom-up” and “top-down”.

In the “bottom-up” method, the source material is loaded under the gradient and mixed with a high-density medium. During centrifugation, particles less dense than the surrounding medium float upwards, and after sufficient time, they reach their fraction corresponding to their own density.

In the “top-down” method, the starting sample is loaded to the top of the low-density medium, and particles then travel into the gradient until they reach their equilibrium density. The type of setting depends on the purpose of the separation [56].

With the isolation method of size exclusion chromatography, EV-containing samples can be distinguished by size. This is because larger particles in the sample will elute faster than smaller particles. It occurs because larger particles cannot enter the column pores, allowing them to pass through the stationary phase more quickly. The separation quality is affected by pore size, column packaging, the ratio of column length to diameter, flow rate, sample volume, and concentration [56]. The longer the stationary phase, the more effective the separation will be. The advantages of this method include sample purity and the fact that the separated EVs are not subjected to strong shear forces, unlike centrifugation. On the other hand, if the vesicle is not perfectly spherical, it may have a different elution time than its regular counterparts with the same mass [58].

*PEG-based precipitation* is a less frequently used procedure, according to these studies, accounting for only 14% [57], but in terms of samples, it provides a better yield with a lack of purity. This reduced purity is due to co-precipitations of proteins and other contaminating particles. This is the biggest drawback of this method but can be addressed with an added purity step, such as filtering or size exclusion chromatography, as well as the ultracentrifugation (UC) technique [59]. On the other hand, it is noteworthy that UC has a greater tendency to form aggregates [60]. The properties of each isolation method are summarized in Table 2.

Extracellular vesicles can be isolated from vegetables, fruits, and medicinal plants. However, the recognition vesicles do not always contain the same constituents as their source of origin [20], so exploring their novel potential applications and effects is worth exploring. Several plant extracellular vesicles have been shown to improve disease progression in certain diseases such as breast cancer [35] and liver carcinoma [22]. Extracts of aloe species are still popular in the treatment of various injuries. In a study with Aloe Saponaria, EVs successfully showed that vesicles can also show promise in the treatment of chronic wound healing [34]. However, it is worth bearing in mind that vesicles isolated from Citrus reticulata Blanco have not been shown to have cytotoxic effects on either cancerous or healthy cells. Still, as they do not damage cells, they may be ideal candidates as drug delivery systems in this case [42]. 

These results suggest that more research is required to clarify and understand the effects and behavior of plant-derived extracellular vesicles.

### 2.3. Extracellular Vesicles Containing Active Pharmaceutical Ingredients

**Figure 4:**
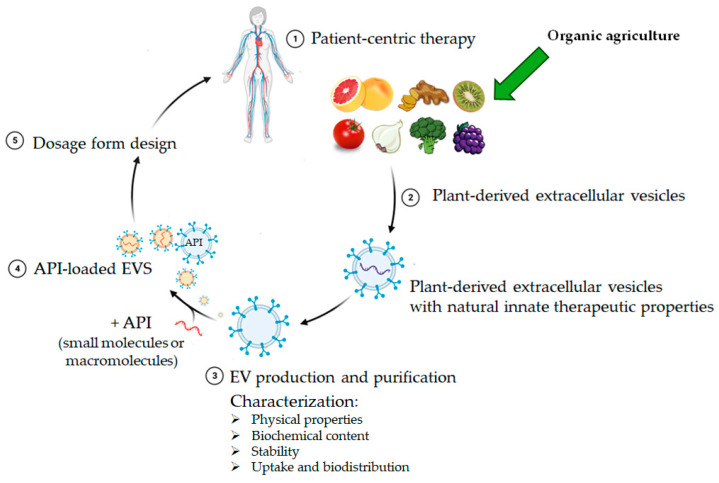
The path of EVs from plant to patient. The red chain represents the external loading of macromolecules, while the purple line indicates the internal macromolecules of the PDEV.

The research on EVs produced by plants as drug delivery systems is an exciting new area of pharmaceutical experimentation. This process is illustrated in Figure 4. EVs from specific plants have already demonstrated suitable properties to serve as ideal candidates for drug delivery systems. These vesicles typically exhibit a size range between 95 and 224 nm and generally maintain an intact spherical structure. Following the encapsulation of drugs, the size of the vesicles has been observed to increase from 113 nm to 329 nm, which is also optimal for cellular uptake. The sources of the vesicles, the encapsulated active ingredients, and their physical properties are summarized in Table 3. 

## 3. Discussion

Interest in plant-derived extracellular vesicles has been growing steadily in recent years. However, the PRISMA flow chart about the systematic search shows that their usage as drug carrier systems is still less widespread. Certain plants suitable for this purpose involved medicinal plants, fruits, and vegetables. Their use is advantageous over mammalian EVs in several aspects. Since their usage causes fewer immune reactions, they are much safer than mammalian EVs. Moreover, their biological active substances can show additional effects on targeted cells. With surface modification, the targeted drug delivery could be improved. Plants are accessible resources for cost-effective and scalable EV isolation. However, scalability is a complex problem to solve because a standardized production method is not yet available at the laboratory or industry levels. Ultracentrifugation is the gold standard method, but sheer force can damage the extracellular vesicles. Furthermore, this method is time-consuming, similar to density gradient centrifugation. However, density gradient centrifugation produces high-purity EVs [63]. This obstacle does not yet allow their use in clinical practice. Thus, a new reproducible, efficient isolation method should be developed soon. None of the currently available production methods can be used alone to produce large quantities and high-purity EVs. Combined steps will undoubtedly reduce the methods’ cost-effectiveness and increase the isolation’s production time [64]. The ideal method produces high-purity EVs in a short period of time and with consistent quality. Solving this problem is critical for practical application, and it is a huge challenge for researchers. Plant sources are cheaper and more easily available, which could significantly reduce procedural costs. It is worth mentioning that plants from natural origins can differ in content material. Therefore, this may affect their EV content and their biological effect. 

Due to the different production methods and diverse sample qualities, formulating a standardized drug delivery system is challenging. An appropriate size range is required to encapsulate the drug in vesicles. If the vesicle size is too small, the drug could be adsorbed onto the vesicle surface, and if it is too large, the drug is less efficiently delivered into the cell. Additionally, a small sample volume makes it difficult to carry out and manage experiments.

In contrast to extracellular vesicles, liposomes are composed of an artificially produced lipid bilayer membrane capable of transporting both hydrophobic and hydrophilic active substances. Due to their artificial origin, they do not contain proteins in their primary state, but liposomes in which proteins can be incorporated can be produced; these are known as proteoliposomes [65].

Even though liposomes do not contain proteins in their primary state, they have a significant advantage over EVs in that there is a large amount of literature on their production and various uses and modifications. In contrast, there is less information available on the use of EVs as drug delivery systems. Although liposomes can be designed with diverse lipid compositions, this variability is not available in plant-derived vesicles due to their natural origin [66]. All in all, this aspect shows that much research is still needed on the everyday use of plant-derived extracellular vesicles. Despite this, research on extracellular vesicles is growing and represents an exciting new area of future drug discovery.

## 4. Materials and Methods 

The Preferred Reporting Items for Systematic Reviews and Meta-Analyses (PRISMA 2020) guidelines were followed to search for the relevant studies and reports.

### 4.1. Eligibility Criteria

The following criteria were established for articles to be eligible for inclusion in this systematic review: Only original research articles published in peer-reviewed journals were included, while reviews, editorials, conference papers, and commentaries were excluded. The search was confined to articles published in English between 2016 and 2024. We did not set criteria to exclude any isolation method by which plant vesicles can be produced. Additionally, no clinical trial limits were imposed, and all in vivo and in vitro studies were considered eligible.

### 4.2. Search Strategy

A systematic search was conducted in PubMed, Ovid Medline, Scopus, ScienceDirect, Embase, and Web of Science to identify relevant articles on plant-derived extracellular vesicles as drug delivery systems using search keywords and their equivalent synonyms. We developed our own search queries as follows: (Plant-derived) AND (Extracellular vesicles) AND (drug delivery). The results were synthesized and presented in tabular form.

### 4.3. Data Collection and Extraction

We used the PRISMA 2020 flow diagram to extract the most relevant data essential for synthesizing the results. First of all, results obtained from all databases were exported to the EndNote reference manager, and duplicate studies were removed. The rest of the articles were screened successfully based on the title, and after that, the rest of the articles were excluded based on the abstract. Then, all eligible articles were reviewed and analyzed. The relevant information was collected and tabulated into the following variables: plant name, components of the vesicles, isolation method, application, size, and appearance.

## 5. Conclusions

Plant-derived extracellular vesicles represent a promising frontier in drug delivery systems, offering numerous advantages over synthetic nanocarriers. Their natural biocompatibility, cost-effectiveness, and potential for large-scale production make them an attractive option for pharmaceutical research and development. PDEVs have demonstrated the ability to enhance drug solubility, bioavailability, and targeted delivery while potentially reducing toxicity and dosage requirements. The synergistic effects between encapsulated drugs and the vesicles’ inherent bioactive compounds open up new possibilities for therapeutic applications. However, challenges remain in standardizing isolation and formulation methods for clinical use. As research in this field progresses, PDEVs are poised to play a significant role in advancing drug delivery technologies, potentially revolutionizing treatment approaches for various diseases while aligning with sustainable and environmentally friendly biotechnological processes. This review underscores the importance of green manufacturing practices in the production of PDENs. This emphasis on sustainability is a novel aspect that sets this review apart from others.

## Figures and Tables

**Table 1:** Characteristics of different types of plant-derived vesicles.

Plant Name	Components of the Vesicles	Isolation Method	Application	Size [nm]	Appearance	Ref.
*Apium* *graveolens* L.	**Lipids:** Diacylglycerol ~45% Phosphatidic acid ∼15% Dehydrophytosphingosine ∼15% Digalactosyl diglyceride ∼13% Monogalactosyldiacylglycerol ∼12% Proteins	Ultracentrifugation + Density gradient centrifugation	Effect against tumor proliferation	111.8	Nearly spherical	[28]
*Malus domestica*, ‘*Royal gala*’	Cyanidin-3 glucoside **miRNA**	Ultracentrifugation + Size exclusion chromatography	Testing biological activity and miRNA carrier potential	253 ± 13	-	[20]
*Punica* *granatum* L.	Cy-3,5diglc **miRNA**	Ultracentrifugation + Size exclusion chromatography	Testing biological activity and miRNA carrier potential	233 ± 2.6	-	[20]
*Allium sativum*	**miRNA** **Proteins** **Amino acids and derivatives** 22.35% **Alkaloids** 9.84% **Lipids** 11.8%: Sphingolipids 1% Lysophosphatidylcholine 1.15% Lysophosphatidylethanolamine 1.41% Free fatty acids 96.36%	Ultracentrifugation + Density gradient centrifugation	Preventing ulcerative colitis	79.60	Cup-shaped morphology with intact membranes	[31,32]
*Allium tuberosum*	**miRNA** **Proteins** **Lipids:** Phosphatidylcholine 43% Phosphatidylethanolamine 33% Monogalactosyl-diacylglycerol 10% Digalactosyldiacylglycerol 6% Phosphatidic acid 3% Phosphatidylglycerol 3%	Differential ultracentrifugation	Effect on neuroinflammation	144 ± 3.3	Round shape	[30,33]
*Aloe Saponaria*	-	PEG-based precipitation	Chronic wound healing	156.2 ± 1.6	Spherical morphology	[34]
*Brassica oleracea var. capitata* L.	-	Ultrafiltration + Size exclusion chromatography	Inflammation and apoptosis inhibition	98.8	Spherical shapes	[18]
*Broccoli*	**miRNA** Sulforaphane Indole-3-carbinol	Ultracentrifugation + Size exclusion chromatography	Testing biological activity	241 ± 8.8	-	[20]
*Brucea javanica*	**Proteins** Bruceine D Brusatol **Lipids:** Triglyceride 78.2% Diglyceride 11.6% Ceramides 3.3% Phosphatidic acid 1.78% Phosphatidylglycerol 1.71%	Differential ultracentrifugation	Treating breast cancer via PAM pathway	104.6 ± 29.4	Cup shape	[35]
*Cannabis sativa* Ark-01	CBD	Differential ultracentrifugation + Density gradient centrifugation	Human hepatocellular carcinoma	163.9 ± 27.16	Spherical or oval-shaped with an intact bilayer membrane	[36]
*Cannabis sativa* Krmn-01	CBD	Differential ultracentrifugation + Density gradient centrifugation	Human hepatocellular carcinoma	133.2 ± 5.75	Spherical or oval-shaped with an intact bilayer membrane	[36]
*Catharanthus roseus*	**Amino acids** **Proteins** **Alkaloids:** Vinpocetine Carbohydrates **Fatty acids** **Lipids:** Ether-phosphatidylcholines 16.55% Phosphatidylglycerols 15.69% Phosphatidylinositol 14.01% Ether-phosphatidylglycerols 9.35% Phosphatidylcholine 7.26% Phosphatidylethanolamine 7.01% Phosphatidic acid 6.34% Other phospholipid derivatives 20.17%	Differential ultracentrifugation + Density gradient centrifugation	Testing the immunomodulatory effect	141.7	Rounded hollow vesicle shape	[37]
*Citrus × limon* (lemon)	Flavonoids: Eriocitrin Quercetin Vicenin-2 Naringin Hesperidin Limonoids: Limonin-17-β-D-glucoside	Electrophoresis combined with dialysis	Gastric cancer	-	Intact vesicles	[38,39]
*Citrus × sinensis tarocco* (orange)	-	Ultracentrifugation	SARS-CoV-2	167 ± 10	Intact membrane with a round shape	[40]
*Citrus reticulata* (red mandarin)	**Sugar** **Proteins** **Lipids** **Flavonoids:** Neohesperidin Sinensetin Nobiletin	PEG-based precipitation	Testing antioxidant and anti-inflammatory abilities	190	Spherical	[16]
*Citrus sinensis* (orange)	Vitamin C Vitamin E Hesperidin **miRNA**	Ultracentrifugation + Size exclusion chromatography	Testing biological activity	167 ± 5.7	-	[20,41]
*Citrus tangerine* (mandarin)	**Proteins:** HSP70 HSP90 Tetraspanins Annexins ABC transporters Glyceraldehyde-3-phosphate Dehydrogenase **Flavonoid:** Tengeretin Diosmetin diglucoside **Organic acid:** Quinic acid Lysophospholipids	Differential centrifugation + Ultracentrifugation	Colon cancer	255 ± 18	Cup or nearly round shapes	[42]
*Citrus* × *paradisi* (grapefruit)	Ascorbic acid Catalase Glutathione	Differential ultracentrifugation	Examining the antioxidant and drug delivery properties	86–125	Round or oval shape	[43,44]
*Cucumis sativus* L.	**Proteins**	Differential ultracentrifugation + Size exclusion chromatography	Improved dermal drug delivery	167 ± 3	Imperfect spherical shape	[45]
*Dendropanax* *morbifera*	-	Differential ultracentrifugation	Effect against cancer-associated fibroblasts	100–200	Nearly spherical	[46]
*Kaempferia parviflora* (Thai black ginger)	5,7-dimethoxyflavone	Differential ultracentrifugation + Size exclusion chromatography	An alternative approach for enhancing gastric cancer therapy	256.8 ± 14.81	Cup shape	[19]
*Kiwi*	**Lipids:** Phosphatidylcholine 49.27% Phosphatidylethanolamine 16.48% Ceramide 12.86% Monogalactosyl diacylglycerols 4.32% Phosphatidic acid 4.29% Digalactosyl diacylglycerols 3.29% Lysophosphatidylcholine 2.86% Phosphatidylinositol 2.53% Phosphatidylglycerol 2.27% Phosphatidylserine 1.19% Sphingoid base-phosphates 62% Sphingoid base 18% Sphingomyelins 10% Ceramide 6% Ceramide phosphate 3% Mannosylinositol phosphorylceramide 1%	Differential ultracentrifugation + Size exclusion chromatography	Hepatocellular carcinoma	224.5 ± 5.2	Typical vesicle structure	[27,47]
*Momordica* *charantia*	**Lipids:** Sphingosine 55.01% Ceramide 11.01% Phosphatidylethanolamines 8.23% Triglyceride 7.46% Phosphatidylcholine 6.36% Diglyceride 5.21% Phosphatidylglycerols 1.79% Zymosteryl 1.41% **Proteins:** Thioredoxin Peroxidase	Differential ultracentrifugation	Treating ulcerative colitis	132.03	Cup shape	[48]
*Morinda officinalis*	**Lipids** 87.62%: Phosphatidylcholine 46.4% Phosphatidylethanolamine 29.06% Phosphatidylserine 7.71% Phosphatidylinositol 3% Phosphatidic acid 9.5% Phosphatidylglycerol 2% RNA Proteins	Enzyme digestion	Promoting miR-155 expression in endothelial cells	65.46 ± 1.74	Typical exosome-like morphology	[17]
*Panax ginseng*	**Ginsenoside derivatives:** Rb1 64.5% Rg1 31.1% Rg3 2.3% R1 2.0% Rg5 0.1%	Density gradient centrifugation	EVs’ effects on osteoclast differentiation	71.42	Spherical with a lipid bilayer membrane	[49]
*Panax notoginseng* (root)	**miRNA** **Proteins** **Lipids:** Ceramide 26.4% Phosphatidic acid 21.9% Diglyceride 13.1% Triglyceride 12.8%	Ultracentrifugation + Density gradient centrifugation	Treating cerebral ischemia/reperfusion injury	151.3	Spherical	[50]
*Petasites japonicus*	-	Filtration + Differential centrifugation	Elucidating the immunostimulatory effect on dendritic cells	122.6	Spherical	[51]
*Pueraria montana var. lobata*	**Proteins**	Differential centrifugation	Investigating the effects of colitis and their role in the lung inflammatory response	75.51 ± 10.19	-	[52]
*Salvia dominica* (hairy root)	**Proteins:** Cytoskeletal components Chaperon proteins Glycolytic enzymes	Differential ultracentrifugation	Testing anticancer activity	153 ± 1.8	Intact round shape	[53]
*Solanum* *lycopersicum* (tomato)	**Proteins:** Lipoxygenase Alcohol dehydrogenase 2 Protein E8 ATPases Abscisic stress protein 1 **Lipids:** Phosphatidylserine 16% Phosphatidic acid 33% Phosphatidylglycerol 18% Phosphatidylcholine 16% Phosphatidylethanolamine 17%	Differential ultracentrifugation	Examining the antioxidant and drug delivery properties	140–170	Round or oval shape	[43,54]
*Vaccinium* *caesariense* (blueberry)	**Anthocyanin derivatives:** Malvidin Peonidin Petunidin Cyanidin Delphinidin	40.000 rpm Differential ultracentrifugation 100.000 rpm	Immunomodulatory therapy	108.5 ± 2.4 95.7 ± 3.1	Spherical shape	[26]
*Vitis Vinifera* (grape)	**Proteins** **miRNA** **Lipids:** Phosphatidic acids 53.2% Phosphatidylethanolamines 26.1% Phosphatidylcholines 9.03% Phosphatidylinositol 7.43% Mono/di/glycerols 1.86% Phosphatidylserine 1.38%	Differential centrifugation + Ultracentrifugation + Density gradient centrifugation	Effect against acute liver failure	68.06–458.7	Spherical	[22,55]
*Vitis vinifera Kyoho*	-	Centrifugation + Filtering + Ultracentrifugation	Breast cancer	113.1 ± 0.117	Homogenous spheroid structure	[29]
*Zingiber officinale* (ginger)	**Gingerol derivatives** 2.14%	Differential ultracentrifugation	-	70.09 ± 19.24	Saucer-like	[21]

**Table 2:** Advantages and disadvantages of the isolation methods.

	Differential Ultracentrifugation	Size Exclusion Chromatography	PEG-Based Precipitation	Density Gradient Centrifugation
**Advantages**	Widely used and effective Provides good yields	Separate EVs based on size Gentle technique that does not apply strong shear forces	Provides the best yields for EV isolation	Separate EVs based on their density
**Disadvantages**	Lacks specificity in terms of speed and duration of centrifugation Small sample volume capacity May lead to the formation of aggregates	Requires careful optimization of column parameters Limited scalability	May co-precipitate proteins and other contaminants Limited scalability	Requires preparation of density gradient medium The centrifugation step is essential, which may introduce potential artifacts

**Table 3:** Plant-derived extracellular vesicles as drug delivery systems and their properties.

Loaded Drug Name	LogP	Water Solubility [mg/mL]	Molecular Weight [g/mol]	Size Before Loading and After Loading [nm]	Polydispersity	Zeta Potential [mV]	Loading Efficiency	Ref.
Sorafenib (Beyotime China)	4.12 *	<0.01	464.825	224.5 305.9	0.296	−14.9 ± 0.9	EE: 69%	[27]
Metformin Doxorubicin Tamoxifen	−2.6	soluble	129.1636	113.1 ± 0.117 329.8 ± 0.107	NA	−15	NA	[29]
	1.27	10	543.5193					
	5.93 *	0.00102 *	371.5146					
Sodium-thiosulfate (Aladdin Biological Technology Co., Ltd. Shanghai, China)	NA	NA	158.11	113.4 ± 8.9 137.1 ± 10.8	NA	~−4	NA	[61]
Dexamethasone (Wako Osaka, Japan)	1.83	89	392.4611	144 ± 3.3 157 ± 2.8	NA	NA	NA	[30]
Astaxanthin (Sigma-Aldrich St Louis, MO, USA)	7.4 *	0.000667 *	596.852	NA 191.6 ± 2.23	0.166	−15.85 ± 0.92	DL: 6.824%	[14]
Doxorubicin	1.27	10	543.5193	111.8 113.7	NA	−34.24	LE: 87.03%	[28]
Aspirin (Sigma-Aldrich, St. Louis, MO, USA, Cat #5376)	1.18	10	180.1574	108.5 ± 2.4 NA	NA	NA	EE: 36.79%	[26]
Curcumin (Sigma-Aldrich, Cat #C1386)	3.62 *	0.00575 *	368.3799	95.7 ± 3.1 NA	NA	NA	EE: 82.76%	[26]
Curcumin (Sigma Aldrich, St. Louis, MO, USA)	3.62 *	0.00575 *	368.3799	160 ± 3 NA	NA	NA	DL: 3.6% EE: 0.22%	[23]
Tangeretin (Shaanxi, China)	NA	insoluble	372.375	190 220	0.17	−4.8	ER: 71.5 ± 0.19% LC: 4.96 ± 0.22%	[16]

EE—entrapment efficiency; ER—encapsulation rate; LC—loading capacity; LE—loading efficiency; NA—not applicable. Data marked with “*” are estimated properties. Source of properties: [62].

## Data Availability

Not applicable.

## Abbreviations

API—active pharmaceutical ingredients; DGC—density gradient centrifugation; DL—drug loading capacity; DNA—deoxyribonucleic acid; DUC—differential ultracentrifugation; EE—entrapment efficiency; ER—encapsulation rate; EVs—extracellular vesicles; HSP70—70-kDa heat shock protein; LC—loading capacity; LE—loading efficiency; NA—not applicable; PBS—phosphate-buffered saline; PDEVs—plant-derived extracellular vesicles; PEG—polyethylene glycol; PRISMA—Systematic Reviews and Meta-Analyses; RNA—ribonucleic acid; SEC—size exclusion chromatography; UC—ultracentrifugation.
